# Moral distress arising from professionalism dilemmas among medical interns

**DOI:** 10.1590/1806-9282.20260580

**Published:** 2026-10-23

**Authors:** Giulia Francci, Laura Oliveira de Alcântara Ferraz, Marina da Rocha Brito, Rogério Aparecido Dedivitis

**Affiliations:** 1 Centro Universitário Lusíada – Santos (SP), Brazil.

**Keywords:** Moral distress, Medical professionalism, Ethical dilemmas, Medical education

## Abstract

**BACKGROUND:**

Moral distress is defined as a psycho-emotional experience that occurs when individuals recognize the morally appropriate course of action but are unable to carry it out due to external constraints such as institutional hierarchies, organizational norms, or authority pressures. In medical education, this phenomenon is particularly frequent, given the deeply ethical nature of clinical decisions and patient care.

**OBJECTIVE:**

The aim of this study was to translate and adapt the Moral Distress from Professionalism Dilemmas checklist into Brazilian Portuguese.

**METHODS:**

The translation and cross-cultural adaptation process followed internationally recognized guidelines. It included independent translations by two professionals (one with and one without medical training), synthesis of the versions, back-translation, expert committee review, pretesting with 200 medical interns, and final evaluation.

**RESULTS:**

Of the 200 medical students invited, 160 (80%) completed the checklist, with no significant differences by year or gender. Depending on the item, between 0 and 57.5% of participants reported exposure to the professionalism dilemmas investigated. Among exposed students, the intensity of moral distress varied considerably across situations.

**CONCLUSION:**

Moral distress is a prevalent phenomenon in medical education. The adapted checklist demonstrated semantic and conceptual equivalence to the original and may serve as a valuable tool to identify, quantify, and understand moral distress among medical students in clinical training.

## INTRODUCTION

Moral distress is a psycho-emotional experience that arises when an individual recognizes the ethically appropriate action but feels constrained from acting upon it due to external factors such as institutional hierarchies, organizational rules, or pressures from authority figures^1^. In medicine, this phenomenon is frequent because clinical decision-making and patient care are inherently moral activities. The recurrent dissonance between what one believes to be right and what can be done in practice may lead to frustration, guilt, loss of empathy, and, in the long term, burnout^2,3^.

Within this context, what is taught about professional conduct and ethics throughout medical education often becomes a source of tension. Physicians have an ethical commitment to integrity, responsibility, empathy, technical competence, and respect toward patients—principles that should be consistently applied and modeled during clinical training. Medical interns, typically in the final years of undergraduate education, occupy the lowest level in the hospital hierarchy and are therefore in a position of considerable moral vulnerability. They frequently witness unprofessional behaviors by peers or superiors but feel powerless to intervene, question, or even express concern. Such circumstances foster ethical dilemmas and contribute to moral distress, which in turn affects not only interns’ mental health but also their professional and moral development^4^.

Understanding moral distress resulting from professionalism dilemmas experienced by medical interns is therefore essential for improving pedagogical strategies in medical education and for promoting healthier, more ethical, and more formative clinical learning environments.

The Moral Distress from Professionalism Dilemmas checklist^5^ investigates the frequency of events in which ethical values conflict with institutional norms. It addresses topics such as hierarchical relationships, inappropriate behaviors, workplace pressures, privacy concerns, and organizational limitations. By quantifying both the occurrence and the perceived impact of such experiences, the instrument allows for analysis of moral distress patterns and their correlations with social and academic variables. Moreover, it supports the development of pedagogical interventions aimed at fostering ethical, safe, and emotionally sustainable clinical environments.

There is a lack of an instrument adapted specifically for medical interns and, as a result, it is difficult measuring and monitoring educational interventions. Thus, this study aimed to translate and adapt the Moral Distress from Professionalism Dilemmas checklist into Brazilian Portuguese among fifth and sixth-year medical students, to assess moral distress related to professionalism dilemmas during internship training.

## METHODS

This study was previously approved by the institutional review board of the institution where it was performed and all participants who accept the participation signed digitally the free and informed consent form.

Following consent and authorization from the original instrument’s author, the translation and cross-cultural adaptation process was conducted in accordance with established international guidelines^6^. The procedure comprised six sequential stages: (1) initial translation, (2) synthesis of translations, (3) back-translation, (4) expert committee review, (5) pretesting of the pre-final version, and (6) final assessment of the entire adaptation process.

Stage I: Inicial translation: The first stage involved two independent forward translations from English (source language) into Brazilian Portuguese (target language). Each translator had a distinct professional background, allowing for comparison of perspectives, identification of ambiguities, and detection of inadequate wording for further discussion and resolution. Both translators submitted written reports justifying their lexical and syntactic choices and noting any uncertainties or challenging phrases. Translator 1: was a medical professional familiar with the concepts addressed in the instrument, providing a clinically oriented translation. Translator 2: was a professional without a medical background and was not informed about the checklist’s purpose, ensuring a linguistically natural translation unaffected by disciplinary bias.

Stage II: Synthesis of translations: A synthesis meeting was held among both translators and an observer to reconcile discrepancies and produce a single synthesized version (T-12). A report documenting all decisions, discussions, and rationales for the final choices was prepared at the end of this stage.

Stage III: Back-translation: Two translators, native English speakers blind to the original instrument, independently back-translated the synthesized Brazilian Portuguese version (T-12) into English to verify the accuracy and conceptual fidelity of the translation. These translators had no medical background or prior knowledge of the checklist’s content. Two back-translated versions (BT1 and BT2) were produced and compared with the original instrument to detect any conceptual or semantic inconsistencies.

Stage IV: Expert committee review: An expert committee—composed of two health professionals, three undergraduate medical students, two native English translators, and back-translators involved in the process—reviewed all prior documents to reach consensus on discrepancies and ensure conceptual equivalence. The committee’s evaluation focused on four main areas: semantic, idiomatic, experiential, and conceptual correspondence. This review resulted in the pre-final version of the instrument in Brazilian Portuguese. During the expert committee review, particular attention was given to preserving conceptual equivalence while ensuring contextual relevance. Whenever an item was considered potentially inapplicable to Brazilian undergraduate medical training, the committee evaluated whether adaptation, replacement, or exclusion would best preserve the underlying construct. Decisions were based on consensus and aimed to minimize threats to the content validity of the adapted instrument while maintaining maximum comparability with the original version.

Stage V: Pretesting of the pre-final version: The pre-final version of the Moral Distress from Professionalism Dilemmas checklist was administered to fifth and sixth-year medical interns from the institution. Cognitive interviews or debriefing (e.g., think-aloud procedures, comprehension and acceptability probing) were not conducted as pretest, since the content of the checklist was considered intuitive. The instrument was distributed electronically via Google Forms following completion of the informed consent form. A total of 200 students was invited to participate voluntarily. Each participant completed the checklist individually and anonymously in a single sitting. The checklist evaluates two related but distinct constructs. First, participants indicate whether they have been exposed to each professionalism dilemma. Second, for situations that occurred, they rate the intensity of the resulting moral distress using a four-point scale. Thus, frequency of exposure reflects the occurrence of professionalism dilemmas, whereas the distress score reflects participants’ emotional response to those events rather than their frequency. Responses were scored on a four-point moral distress scale: 1—no distress (although it happened); 2—mild distress (slightly uncomfortable during the event, but not much thought afterward); 3—moderate distress (disturbed and distressed during the event, felt helpless to do anything and thought about it for a while afterward, but does not bother me much now); and 4—severe distress (disturbed and distressed during the event, felt helpless to do anything, and still bothers me). There were not domains or subscales in the checklist.

Stage VI: Final evaluation: A comprehensive review of the entire translation and adaptation process was conducted by the authors to confirm linguistic accuracy and conceptual equivalence across all stages.

Statistical analysis: Categorical variables were described using frequency distributions (number and percentage), while numerical data were analyzed using measures of central tendency (mean and median) and variability (range and standard deviation). To compare total checklist scores between fifth and sixth-year medical students, the Mann-Whitney U test was applied. Associations between total scores, academic year, and gender were assessed using the Kruskal-Wallis test. A boxplot was generated to visually represent associations between numerical and categorical variables. A 5% significance level was adopted for all statistical tests. All analyses were performed using Stata Statistical Software, version 19^7^. In the comparison between the groups of students, the total score with the average of item responses were considered.

## RESULTS

During the process of translation and cultural adaptation of the checklist, several minor adjustments were made to ensure greater clarity and appropriateness to the Brazilian academic and cultural context.

The main adaptation involved substituting the term “resident” with “intern” (items S3 and S53), as the target population of this study comprised undergraduate medical interns rather than postgraduate residents. Similarly, “medical residency” was translated as “medical internship” (item S4) to maintain contextual coherence.

The expressions “senior physicians” were adapted to “supervisors” (items S11 and S20) or “medical supervisors” (item S29) to better reflect the hierarchical and educational structures of Brazilian medical training.

Additional lexical refinements were made to improve readability and conceptual precision. For instance, “alcohol” was rendered as “alcoholic beverages” (item S6), and “being caught” was adapted to “being found” (item S7). The term “nepotism” was replaced by “favoritism in scheduling or providing medical services to relatives or friends” (item S44) to prevent potential ambiguity. Similarly, “red envelope”—a culturally specific expression—was adapted to “gratuities” (item S47) to achieve Brazilian cultural equivalence.

Item S18, which addresses “salary and benefits,” was excluded because this situation is not applicable to undergraduate medical interns in Brazil, who do not receive salaries or employment-related benefits during their clinical training. Rather than reflecting a cultural or linguistic difference, this exclusion results from a structural characteristic of Brazilian medical education, in which internship constitutes a compulsory curricular component rather than an employment relationship. The expert committee considered retaining the item with modified wording; however, any adaptation would substantially alter its original construct, potentially compromising content validity. Therefore, exclusion was judged to be the most appropriate strategy to preserve conceptual integrity of the remaining instrument. Consequently, the Brazilian version contains one fewer item than the original checklist, and direct score comparisons with studies using the original instrument should take this structural difference into account. Finally, items S23 and S48 underwent minor linguistic adjustments for clarity, without altering their original meaning (Supplementary 1).

Among the 200 students invited to participate, 160 (80%) completed the checklist. Depending on the checklist item, between 0 and 57.5% of participants reported having experienced the situations described. Among those exposed to each situation, the intensity of moral distress varied according to the four-point response scale, with substantial heterogeneity across items (Figure 1).

**Figure 1:**
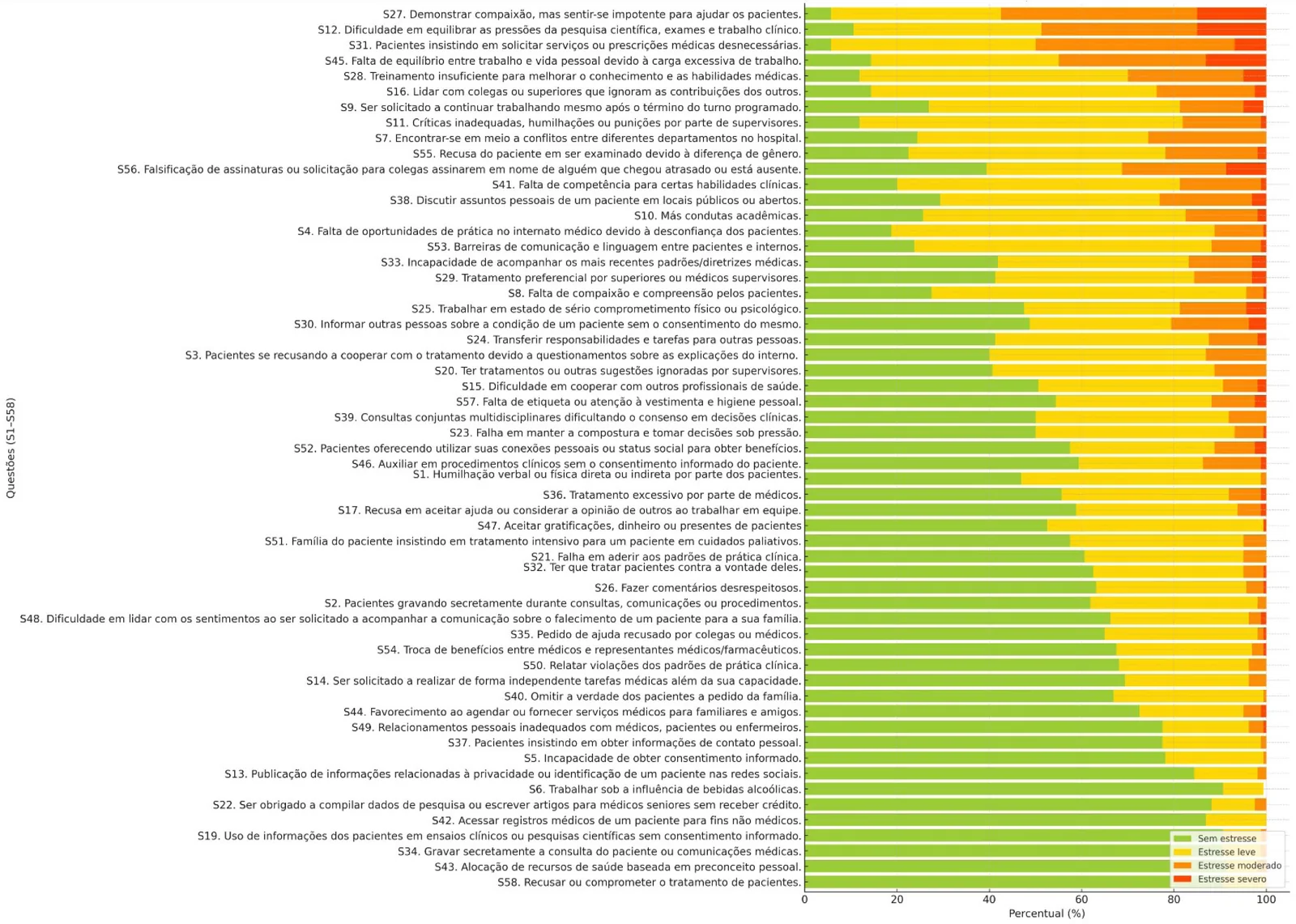
Intensity of moral distress related to professionalism dilemmas.

When comparing results by academic year (fifth vs. sixth year) and gender, no statistically significant differences were observed (Table 1).

**Table 1:** Total questionnaire score according to academic year (fifth and sixth year) and gender.

Score measures	Total	Fifth year		Sixth year		p-value
		Male	Female	Male	Female	
		Measures				
n	160	31	58	24	47	0.847
Range	92–249	142–249	148–249	159–229	92–220	
Median	186	184.0	182.5	188.5	187.0	
Mean	185.8	184.8	185.6	188.2	185.7	
Standard deviation	22.2	23.3	21.7	18.3	24.4	

p-value obtained using the Kruskal-Wallis test.

## DISCUSSION

Analysis of the 57 situations included in the Moral Distress from Professionalism Dilemmas checklist revealed that moral distress is present among medical interns, although with varying intensities. The predominant categories reported were mild stress (38%) and moderate stress (34%), while no stress accounted for approximately 18% and severe stress for around 10% of responses. This pattern suggests that, even though not all situations provoke intense suffering, a consistent background level of moral discomfort permeates students’ clinical experiences.

It is important to distinguish between exposure to professionalism dilemmas and the emotional impact resulting from those experiences. Frequent exposure does not necessarily produce severe moral distress, and conversely, relatively uncommon events may generate intense moral suffering when they involve profound ethical conflicts. Therefore, the checklist should be interpreted as assessing both the occurrence of ethically challenging situations and the magnitude of the distress they provoke, rather than assuming a direct relationship between frequency and emotional impact.

The exclusion of item S18 deserves consideration when interpreting cross-cultural equivalence. Although the adaptation preserved the conceptual domains explored by the original checklist, one employment-related situation could not be meaningfully represented in the Brazilian undergraduate context. Consequently, conceptual equivalence was maintained at the instrument level, whereas strict structural equivalence was slightly modified by reducing the total number of items. This difference is unlikely to affect the assessment of professionalism-related moral distress among Brazilian medical interns because the excluded scenario is absent from their educational reality. Nevertheless, future international comparative studies should acknowledge this difference when comparing total scores or psychometric properties across versions, preferably using standardized or item-level analyses rather than raw total scores.

Clinical care involves the interaction of multiple moral agents—including patients, families, and healthcare professionals—each of whom holds personal perspectives on what is right or wrong, desirable or undesirable, and assigns relative weight to these moral values^1^. The moral distress experienced by healthcare professionals typically escalates during crises and diminishes once the precipitating event is resolved. However, a moral residue remains, contributing to an elevated baseline level of distress that amplifies during subsequent crises^8^.

The concept of moral distress is relatively recent, with approximately three decades of scholarly discussion. Most works on the topic cite Jameton, who introduced the term to distinguish moral distress from other types of ethical problems in healthcare. Situations that generate moral distress are those in which the professional clearly understands which moral principle applies but is constrained from acting accordingly. When uncertainty exists regarding which principle should guide action, the situation constitutes moral uncertainty rather than moral distress. Furthermore, moral distress differs from a moral dilemma: in the former, the individual knows the correct action but is unable to perform it; in the latter, two or more moral principles conflict, each supporting different actions^9^.

The situations that elicited the highest levels of moderate to severe stress in this study were those related to medical hierarchy, ethical limitations in clinical practice, and conflicts of values—such as verbal humiliation, lack of autonomy in decision-making, and pressure to follow directives contrary to one’s moral convictions. These findings are consistent with those of Song et al., who also identified hierarchical and authority-related dilemmas as leading sources of moral distress among medical trainees.

The comparative analysis revealed no significant associations between moral distress levels and gender or academic year, suggesting that distress is shaped more by shared institutional contexts than by individual demographic factors. Similar findings have been reported among medical residents, who, despite being at different training stages, share similar perceptions regarding professional and unprofessional conduct, indicating that hospital environments tend to homogenize moral perceptions^4^.

Conversely, women approach moral issues through an ethic of care, whereas men are guided more by principles of justice^10^. This distinction could theoretically influence perceptions of moral distress. However, neuroimaging research demonstrated that, although there are neural differences between genders in moral sensitivity, these do not necessarily translate into differences in the intensity of moral distress^11^. Therefore, the absence of gender-related differences in the present study may reflect the influence of modern medical curricula that emphasize integrative learning and socio-emotional competencies, which tend to mitigate gender disparities in moral and emotional formation.

Moral distress is not an inevitable consequence of hierarchical position. Factors related to the doctor–patient relationship and the intrinsic motivation to perform good clinical work were negatively associated with distress, suggesting potential protective effects. Positive professional role models may help students cultivate resilience and moral integrity, mitigating the effects of ethically challenging situations^2^.

Some items yielded unexpected results. In item S13, few students reported posting patient-related information on social media—a surprisingly low frequency given the widespread use of digital platforms. This may indicate higher ethical awareness among students or potential underreporting due to social desirability bias.

In item S20, which addressed the perception of supervisors ignoring students’ suggestions, it was hypothesized that this would be a major source of moral distress given the traditional hierarchical structure of healthcare teams. However, most participants did not report feeling ignored, suggesting increased openness and dialog between supervisors and students. This may reflect evolving teaching dynamics, where preceptors encourage shared decision-making and recognize the educational value of participatory learning environments.

Similarly, in item S23, it was expected that students would report significant difficulty maintaining composure or making decisions under pressure due to limited clinical experience. Surprisingly, most participants reported feeling relatively confident in such situations. This may indicate the development of coping strategies and decision-making confidence throughout training, though self-perceived competence may not always reflect actual clinical ability.

This study presents certain limitations. Data were collected from a single institution, which may limit generalization. Although participation was voluntary and anonymous, response bias cannot be excluded. Given the sensitive topic, underreporting is possible. Online administration could be related to potential selection bias and response-condition effects. The checklist included 57 items reflecting contemporary professional dilemmas; different scenarios might yield varying responses. Moreover, no statistical correction for multiple comparisons was applied, as the analysis had an exploratory nature. Thus, some associations that appeared significant may be spurious. Finally, participants’ reports of observed or experienced behaviors may not predict future professional actions. A multicenter validation is a key next step.

The findings underscore the need for medical schools to implement structured strategies that promote ethical reflection, empathic supervision, and psychological support during internship training. Incorporating formal spaces for the discussion of ethical dilemmas and encouraging open communication with mentors may reduce moral distress and strengthen professional identity formation in medical students.

## CONCLUSION

More than 70% of medical interns reported experiencing some degree of moral distress, with approximately 44% describing moderate or severe stress in response to ethical or behavioral dilemmas. This prevalence suggests that moral distress is a significant reality in medical training. By quantifying experiences of moral distress, educators and institutions can better recognize the ethical tensions inherent in medical education and design targeted pedagogical interventions to promote emotional well-being, professional integrity, and resilience among trainees.

## Data Availability

The datasets generated and/or analyzed during the current study are available from the corresponding author upon reasonable request.
