# Supplementary material for: Moral distress arising from professionalism dilemmas among medical interns
Source: Rev Assoc Med Bras (1992). 2026 Oct 23;72(10):e20260580. doi: 10.1590/1806-9282.20260580 (PMC13693302; doi:10.1590/1806-9282.20260580)
Supplement: Supplementary file 1 [file 1806-9282-ramb-72-10-e20260580-suppl1.doc]

**Supplementary 1.** Translated version of the “Moral Distress from Professionalism Dilemmas” questionnaire into Brazilian Portuguese.

S1. Humilhação verbal ou física direta ou indireta por parte dos pacientes.

S2. Pacientes gravando secretamente durante consultas, comunicações ou procedimentos.

S3. Pacientes se recusando a cooperar com o tratamento devido a questionamentos sobre as explicações do interno.

S4. Falta de oportunidades de prática no internato médico devido à desconfiança dos pacientes.

S5. Incapacidade de obter consentimento informado.

S6. Trabalhar sob a influência de bebidas alcoólicas.

S7. Encontrar-se em meio a conflitos entre diferentes departamentos no hospital.

S8. Falta de compaixão e compreensão pelos pacientes.

S9. Ser solicitado a continuar trabalhando mesmo após o término do turno programado.

S10. Más condutas acadêmicas.

S11. Críticas inadequadas, humilhações ou punições por parte de supervisores.

S12. Dificuldade em equilibrar as pressões da pesquisa científica, exames e trabalho clínico.

S13. Publicação de informações relacionadas à privacidade ou identificação de um paciente nas redes sociais.

S14. Ser solicitado a realizar de forma independente tarefas médicas além da sua capacidade.

S15. Dificuldade em cooperar com outros profissionais de saúde.

S16. Lidar com colegas ou superiores que ignoram as contribuições dos outros.

S17. Recusa em aceitar ajuda ou considerar a opinião de outros ao trabalhar em equipe.

S19. Uso de informações dos pacientes em ensaios clínicos ou pesquisas científicas sem consentimento informado.

S20. Ter tratamentos ou outras sugestões ignoradas por supervisores.

S21. Falha em aderir aos padrões de prática clínica.

S22. Ser obrigado a compilar dados de pesquisa ou escrever artigos para médicos seniores sem receber crédito.

S23. Falha em manter a compostura e tomar decisões sob pressão.

S24. Transferir responsabilidades e tarefas para outras pessoas.

S25. Trabalhar em estado de sério comprometimento físico ou psicológico.

S26. Fazer comentários desrespeitosos.

S27. Demonstrar compaixão, mas sentir-se impotente para ajudar os pacientes.

S28. Treinamento insuficiente para melhorar o conhecimento e as habilidades médicas.

S29. Tratamento preferencial por superiores ou médicos supervisores.

S30. Informar outras pessoas sobre a condição de um paciente sem o consentimento do mesmo.

S31. Pacientes insistindo em solicitar serviços ou prescrições médicas desnecessárias.

S32. Ter que tratar pacientes contra a vontade deles.

S33. Incapacidade de acompanhar os mais recentes padrões/diretrizes médicas.

S34. Gravar secretamente a consulta do paciente ou comunicações médicas.

S35. Pedido de ajuda recusado por colegas ou médicos.

S36. Tratamento excessivo por parte de médicos.

S37. Pacientes insistindo em obter informações de contato pessoal.

S38. Discutir assuntos pessoais de um paciente em locais públicos ou abertos.

S39. Consultas conjuntas multidisciplinares dificultando o consenso em decisões clínicas.

S40. Omitir a verdade dos pacientes a pedido da família.

S41. Falta de competência para certas habilidades clínicas.

S42. Acessar registros médicos de um paciente para fins não médicos.

S43. Alocação de recursos de saúde baseada em preconceito pessoal.

S44. Favorecimento ao agendar ou fornecer serviços médicos para familiares e amigos.

S45. Falta de equilíbrio entre trabalho e vida pessoal devido à carga excessiva de trabalho.

S46. Auxiliar em procedimentos clínicos sem o consentimento informado do paciente.

S47. Aceitar gratificações, dinheiro ou presentes de pacientes.

S48. Dificuldade em lidar com os sentimentos ao ser solicitado a acompanhar a comunicação sobre o falecimento de um paciente para a sua família.

S49. Relacionamentos pessoais inadequados com médicos, pacientes ou enfermeiros.

S50. Relatar violações dos padrões de prática clínica.

S51. Família do paciente insistindo em tratamento intensivo para um paciente em cuidados paliativos.

S52. Pacientes oferecendo utilizar suas conexões pessoais ou status social para obter benefícios.

S53. Barreiras de comunicação e linguagem entre pacientes e internos.

S54. Troca de benefícios entre médicos e representantes médicos/farmacêuticos.

S55. Recusa do paciente em ser examinado devido à diferença de gênero.

S56. Falsificação de assinaturas ou solicitação para colegas assinarem em nome de alguém que chegou atrasado ou está ausente.

S57. Falta de etiqueta ou atenção à vestimenta e higiene pessoal.

S58. Recusar ou comprometer o tratamento de pacientes.
